# The Viral G Protein-Coupled Receptor ORF74 Hijacks β-Arrestins for Endocytic Trafficking in Response to Human Chemokines

**DOI:** 10.1371/journal.pone.0124486

**Published:** 2015-04-20

**Authors:** Sabrina M. de Munnik, Albert J. Kooistra, Jody van Offenbeek, Saskia Nijmeijer, Chris de Graaf, Martine J. Smit, Rob Leurs, Henry F. Vischer

**Affiliations:** Department of Chemistry and Pharmaceutical Sciences, Division of Medicinal Chemistry, Amsterdam Institute for Molecules, Medicines and Systems (AIMMS), VU University Amsterdam, Amsterdam, The Netherlands; Loyola University Chicago, Stritch School of Medicine, UNITED STATES

## Abstract

Kaposi’s sarcoma-associated herpesvirus-infected cells express the virally encoded G protein-coupled receptor ORF74. Although ORF74 is constitutively active, it binds human CXC chemokines that modulate this basal activity. ORF74-induced signaling has been demonstrated to underlie the development of the angioproliferative tumor Kaposi’s sarcoma. Whereas G protein-dependent signaling of ORF74 has been the subject of several studies, the interaction of this viral GPCR with β-arrestins has hitherto not been investigated. Bioluminescence resonance energy transfer experiments demonstrate that ORF74 recruits β-arrestins and subsequently internalizes in response to human CXCL1 and CXCL8, but not CXCL10. Internalized ORF74 traffics via early endosomes to recycling and late endosomes. Site-directed mutagenesis and homology modeling identified four serine and threonine residues at the distal end of the intracellular carboxyl-terminal of ORF74 that are required for β-arrestin recruitment and subsequent endocytic trafficking. Hijacking of the human endocytic trafficking machinery is a previously unrecognized action of ORF74.

## Introduction

β-arrestins regulate the magnitude and duration of GPCR signaling by receptor desensitization and internalization. GPCR kinases (GRKs) or the second messenger-dependent protein kinases A (PKA) and PKC phosphorylate serine (S) and threonine (T) residues within the carboxyl-terminal (C-tail) and/or intracellular loops of GPCRs and induce β-arrestin recruitment [1]. β-arrestins remove GPCRs from the cell surface onto early endosomes by linking GPCRs to proteins from the endocytotic machinery and GPCRs are subsequently sorted either to recycling endosomes to mediate resensitization or to late endosomes and lysosomes to facilitate protein degradation [2].

ORF74 is a viral GPCR (vGPCR) encoded by Kaposi’s sarcoma-associated herpesvirus (KSHV). This vGPCR has been linked to Kaposi’s sarcoma (KS) [3], a tumor characterized by proliferating spindle-shaped tumor cells and elevated expression of growth factors and inflammatory mediators. ORF74 shows highest sequence identity to human chemokine receptor CXCR2. Whereas human chemokine receptors predominantly signal via Gα_i_ proteins, ORF74 promiscuously couples to multiple G protein subtypes and activates several cellular signaling proteins including phospholipase C (PLC) [4], NFAT [5] and several MAPK family members [6, 7]. For example, ORF74-induced ERK and Akt phosphorylation is both Gα_i_- and PKC-dependent [7]. In contrast to its human counterpart, ORF74 is constitutively active but binds various human chemokines that modulate this basal activity [4]. These chemokines display different efficacies and include CXCL1 (full agonist), CXCL8 (low potency agonist) and CXCL10 (inverse agonist) [8].

The kinases PKC, GRK5 and GRK6 attenuate ORF74-induced PLC activation, cell proliferation and foci formation [9, 10]. Furthermore, ORF74 is constitutively internalized by interacting with the clathrin-coated vesicle component AP-2 [11, 12]. However, despite the role of β-arrestin in desensitization, internalization and endocytic trafficking of human GPCRs, an interaction between the constitutively active viral ORF74 and intracellular β-arrestin has not been reported. In this study we show that ORF74 recruits both β-arrestin1 and β-arrestin2 in response to CXCL1 and CXCL8 and subsequently internalizes and traffics via endosomes in a β-arrestin-dependent manner.

## Materials and Methods

### Materials

Dulbecco's modified Eagle's medium, trypsin, fetal bovine serum (FBS), Hank’s balanced salt solution (HBSS), penicillin and streptomycin (P/S) were purchased from PAA Laboratories GmbH (Paschen, Austria). Earle’s inositol-free minimal essential medium was obtained from Gibco (Paisley, United Kingdom)). ^125^I-Na (17.4 Ci/mg), ^125^I-CXCL10 (2200 Ci/mmol) and *myo*-[2-^3^H]inositol (1 mCi/ml) were purchased from PerkinElmer Life Sciences (Boston, MA, USA). Chemokines were obtained from PeproTech (Rocky Hill, NJ, USA). The rabbit polyclonal antibody recognizing ORF74 was a kind gift of Dr. Hayward (Johns Hopkins University, Baltimore, MD, USA) [13]. The monoclonal anti-β-arrestin1/2 antibody (clone D24H9, product number 6474) and the STAT3 antibody (clone 79D7, product number 4904) were from Cell Signaling Technology (Boston, MA, USA). The anti-rabbit HRP-conjugated secondary antibody was obtained from Bio-Rad Laboratories (Hercules, CA, USA). Coelenterazine-h was obtained from Promega (Madison, WI, USA). Polyethylenimine (PEI) for transfection was purchased from Polysciences (Warrington, PA, USA). For siRNA transfection experiments, Dharmacon On-target plus non-targeting control siRNA, On-target plus β-arrestin1 and β-arrestin2 siRNA SmartPool were purchased from Thermo Scientific (Epsom, UK). Lipofectamine 2000 was purchased from Invitrogen (Paisley, UK). Poly-L-lysine and cycloheximide were obtained from Sigma-Aldrich (St. Louis, MO, USA).

### Cell culture and transfection

HEK293T cells were cultured at 37°C and 5% CO_2_ in DMEM supplemented with 10% FBS and 1% P/S. Cells were transfected using linear PEI (molecular weight of 25 kDa). Briefly, a total amount of 5 μg DNA (adjusted with empty pcDEF_3_) was diluted in a total volume of 250 μl NaCl solution (150 mM). Next, 250 μl NaCl solution containing 30 μg PEI was added to the DNA solution, vortexed and incubated for 20 min at 22°C. The mixture was added dropwise to the medium of adherent HEK293T cells. In β-arrestin knockdown experiments, HEK293T cells (plated in a 6 well plate) were transfected with a total amount of 4 μg DNA (adjusted with empty pcDEF_3_) and 100 nM siRNA against both β-arrestin1 and β-arrestin2 (1:1) using lipofectamine 2000 according to the manufacturer’s protocol.

### DNA constructs

The cDNA of ORF74 (GenBank accession number U71368 with a silent G to T mutation at position 927) was a gift from Dr. Schwartz (University of Copenhagen, Denmark) and inserted into pcDEF_3_ (a gift from Dr. Langer, Robert Wood Johnson Medical School, Piscataway, NJ, USA). β-arrestin1 and β-arrestin2 C-terminally fused in frame to enhanced yellow fluorescence protein (eYFP) were previously described [14]. ORF74-Rluc8 was generated as previously described [15]. Briefly, the stop-codon of ORF74 was substituted with a SpeI/NotI linker by PCR and fused in frame with Rluc8, which was a kind gift from Dr. Javitch (Columbia University, New York, NY, USA) [16]. The ORF74-R^3.50^A mutant (Ballesteros-Weinstein numbering [17]) was constructed by PCR-based site-directed mutagenesis using the sense primer 5’-cagtctagtggcgtacctcctg-3’ and the anti-sense primer 5’-caggaggtacgccactagactg-3’ and re-introduced into pcDEF_3_. DNA fragments encoding the C-tail of ORF74 containing the different S/T to A mutations were synthesized by Eurofins (Ebersberg, Germany) and introduced into WT-ORF74-pcDEF_3_ using the internal restriction site Eam1105I/AhdI. The Venus-K-Ras, Venus-Rab5a, Venus-Rab7a and Venus-Rab11 constructs were a kind gift from Dr. Lambert (Georgia Health Sciences University, Augusta, GA, USA) [18, 19]. All generated constructs were verified by sequencing.

### Cell surface receptor expression ELISA

Transiently transfected HEK293T cells were seeded in poly-L-lysine-coated 96 well plates and grown overnight. 48h post-transfection, cells were fixed for 5 min with 4% formaldehyde in Tris-buffered saline (TBS: 150 mM NaCl, 50 mM Tris-HCl, pH 7.5). Next, cells were incubated with blocking buffer (1% fat-free milk diluted in 0.1 M NaHCO_3_ (pH 8.6)) for 4h at 22°C, prior to the overnight incubation at 4°C with anti-ORF74 antibody in 0.1% BSA/TBS. The next day, cells were washed three times with TBS and subsequently incubated for 2h at 22°C with HRP-conjugated secondary antibody in blocking buffer. After washing the cells three times with TBS, OPD substrate solution (2 mM o-phenylenediamine (Sigma-Aldrich) in 35 mM citric acid, 66 mM Na_2_HPO_4_, 0.015% H_2_O_2_, pH 5.6) was added to the cells. The enzymatic reaction was terminated by adding H_2_SO_4_ (1 M) and absorbance (490 nm) was measured in a PowerWave plate reader (BioTek).

### Radioligand binding experiments

^125^I-CXCL8 was labeled as previously described [7]. Briefly, 5 μg human CXCL8 was incubated with 0.5 mCi ^125^I-Na in 35 μl Labeling buffer (125 mM Tris-HCl pH 6.8, 150 mM NaCl) in Iodo-Gen coated tubes (Thermo Fisher Scientific) for 12 min at 22°C. ^125^I-labeled CXCL8 was separated from free iodine using a PD-10 column (GE Healthcare). Incorporation of ^125^I and specific activity were subsequently determined using trichloroacetic acid protein precipitation. 24h post-transfection, transiently transfected HEK293T cells were seeded in poly-L-lysine coated 48 well plates. The next day, binding was performed on whole cells by incubating the cells for 3h at 4°C with 100 pM ^125^I-CXCL8 or ^125^I-CXCL10 in binding buffer (50 mM HEPES (pH 7.4), 1 mM CaCl_2_, 5 mM MgCl_2_, 0.5% bovine serum albumin) in the absence or presence of various concentrations unlabeled chemokine. After incubation, cells were washed three times with ice-cold binding buffer supplemented with 0.5 M NaCl and subsequently lysed and counted in a Wallac Compugamma counter.

### Phospholipase C activation assay

24h post-transfection, transiently transfected HEK293T cells were seeded in poly-L-lysine coated 48 well plates and labeled overnight with *myo*-[2-^3^H]-inositol (1 μCi/ml) in Earle's inositol-free minimal essential medium supplemented with 10% FBS and 1% P/S. The next day, cells were washed with buffer (20 mM HEPES, 140 mM NaCl, 5 mM KCl, 1 mM MgSO_4_, 1 mM CaCl_2_, 10 mM glucose) supplemented with 0.1% BSA and incubated for 2h in the above mentioned buffer containing 10 mM LiCl in the absence or presence of indicated concentrations chemokines. Incubation was terminated by placing the cells on ice and aspirating the stimulation buffer prior to the addition of ice-cold 10 mM formic acid. After incubating the formic acid for 90 min on ice, generated [^3^H]-inositol phosphates (InsP) were isolated by anion-exchange chromatography (Dowex AG1-X8 columns; Bio-Rad) and counted by a Packard Tri-Carb liquid scintillation analyzer.

### β-arrestin recruitment and endocytic trafficking bioluminescence resonance energy transfer (BRET)

HEK293T cells were transiently transfected with cDNA coding for wild type (WT) or mutant ORF74-Rluc8 in combination with β-arrestin1-eYFP, β-arrestin2-eYFP or Venus-tagged K-Ras, Rab5a, Rab7a or Rab11 in a 1:4 ratio, in the presence or absence of siRNA targeting β-arrestin1 and β-arrestin2 or control siRNA. 24 hours post-transfection, cells were seeded in white 384-well plates (β-arrestin recruitment) or poly-L-lysine-coated white 96-well plates (endocytic trafficking). The next day, cells were washed with HBSS and incubated with fresh HBSS supplemented with 0.1% BSA. Fluorescence was measured on a Mithras LB940 multilabel plate reader (Berthold Technologies) to monitor expression of eYFP- or Venus-tagged proteins (excitation 485 nm; emission 535 nm). To measure β-arrestin recruitment, cells were incubated with increasing concentrations of chemokines for 10 min at 37°C. Next, 5 μM coelenterazine-h substrate was added and the cells were incubated for an additional 5 min at 37°C. When co-stimulated, cells were pre-incubated for 15 min with 100 nM CXCL10 prior to the addition of 10 nM CXCL1. For kinetic endocytic trafficking experiments, cells were incubated with 5 μM coelenterazine-h substrate for 5 min at 37°C and BRET (emission 540 nm) and Rluc8 luminescence (emission 480 nm) were immediately measured on a Mithras LB940 multilabel plate reader. Then, 100 nM chemokine in the presence of 10 μg/ml cycloheximide was added and the measurements were continued for 60 min. BRET ratios (540/480 emission) were calculated.

### SDS-PAGE and Western blot

48h post-transfection, cells were lysed in RIPA-buffer supplemented with α-complete protease inhibitor cocktail (La Roche), sonicated and protein concentrations were determined using BCA total protein determination kit (Thermo Fisher Scientific). Equal amounts of protein were resolved by SDS-PAGE analysis using 10% gels. After electrophoresis, proteins were transferred to nitrocellulose membranes (Bio-Rad) and subsequently blocked for 1h at 22°C in 5% non-fat milk in 0.1% Tween-20/TBS solution. Then, membranes were incubated overnight at 4°C with indicated primary antibody in 5% BSA in 0.1% Tween-20/TBS solution. The next day, membranes were incubated with HRP-conjugated secondary antibody in blocking buffer for 1h at 22°C. Immunoblots were developed using enhanced chemiluminescence solution (Thermo Fisher Scientific).

### Construction of the model of active β-arrestin1 bound to the ORF74 C-tail

The crystal structure of active β-arrestin1 bound to the C-tail of the V2 vasopressin receptor (V_2_R) (PDB-code 4JQI) [20] was used as template to construct a homology model of the ORF74 C-tail bound to β-arrestin1. First, a manual sequence alignment of the C-terminal residues of V_2_R, CCR5, CXCR2 and ORF74 (starting at position 7.49 according to the Ballesteros-Weinstein numbering scheme [17]) was created. This alignment focused on S and T residues as putative phosphorylation sites, but also on aspartate (D) and glutamate (E) residues as the latter two can act as phosphomimics [21, 22]. Next, a homology model was built with MOE [23] using standard settings in which the distal 19 C-terminal residues of ORF74 (residues 324–342) were inserted into the β-arrestin1 structure using the V_2_R peptide as a template. Subsequent iterative runs of flexible peptide docking using the Rosetta FlexPepDock webserver [24, 25] (generating 300 high resolution structures per run without constraints) and manual refinement with MOE resulted in the final model. FlexPepDock does not take water molecules into account during docking, but the V_2_R-peptide-coupled β-arrestin1 complex shows water-mediated H-bonds between phosphorylated S/T residues from V_2_R and key residues in β-arrestin1. Therefore, water molecules present within 6Å of the V_2_R peptide in the crystal structure were incorporated in the β-arrestin1-ORF74 complex and minimized while keeping the protein and peptide structure rigid.

### Data analysis

Sigmoidal concentration-response curves and radioligand displacement curves were plotted using GraphPad Prism 6 software (GraphPad Software, San Diego). pEC_50_ and IC_50_ values were determined by nonlinear regression. The IC_50_ values obtained from the radioligand displacement curves were converted to K_i_ values using the method of Cheng and Prusoff [26]. Statistical analyses were performed using Graphpad Prism 6 software.

## Results

### ORF74 recruits β-arrestin1 and β-arrestin2 in response to human chemokines

**Fig 1:**
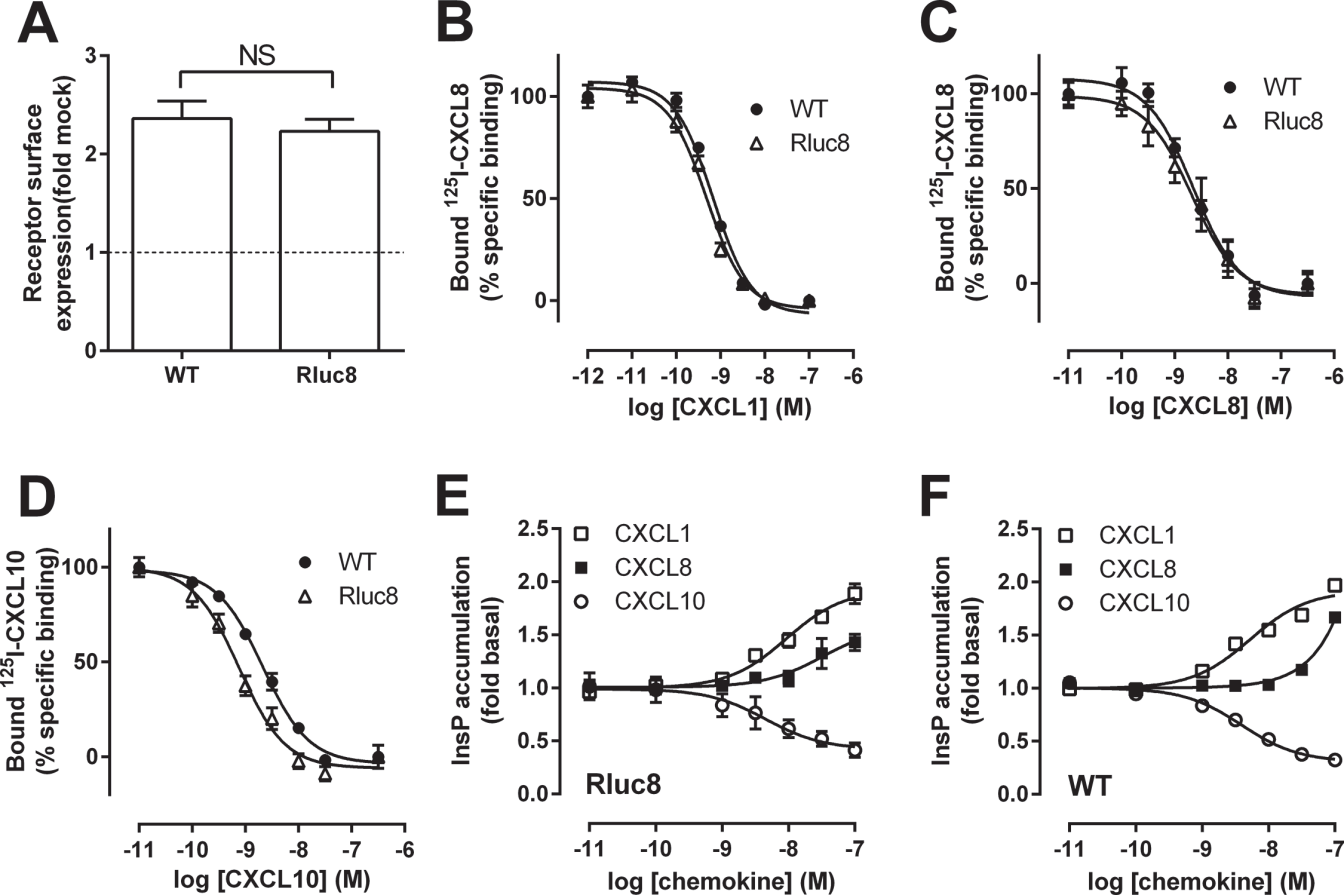
Characterization of ORF74-Rluc8. HEK293T cells were transiently transfected with WT-ORF74 (WT), ORF74-Rluc8 (Rluc8) or empty vector DNA (mock-transfected). (A) Cell surface expression was determined by ELISA. (B-D) Whole cell binding experiments with 100 pM ^125^I-CXCL8 (B, C) or ^125^I-CXCL10 (D) were performed in the presence of increasing concentrations unlabeled CXCL1 (B), CXCL8 (C) or CXCL10 (D). HEK293T cells transfected with ORF74-Rluc8 (E) or WT-ORF74 (F) were stimulated with increasing concentrations of CXCL1 (open squares), CXCL8 (filled squares) or CXCL10 (open circles) and InsP accumulation was quantified. Data are shown as the mean ± SEM of at least three independent experiments each performed in triplicate and are presented as fold over mock-transfected cells (dotted line) (A), percentage of specific ^125^I-CXCL8 (B, C) or ^125^I-CXCL10 binding (D) or fold over basal (E, F). Statistical differences in cell surface expression (A) were determined by a Student t test. NS = not significant.

Fusion of Rluc8 to the C-terminus of ORF74 did not affect cell surface expression of ORF74 (Fig 1A) or binding affinities (Table 1) for CXCL1 (Fig 1B) and CXCL8 (Fig 1C). However, CXCL10 displayed a 3.2-fold higher affinity for ORF74-Rluc8 than for WT-ORF74 (Fig 1D). ORF74-Rluc8 constitutively activated PLC and responded to chemokines (Fig 1E) with similar potencies and efficacies as WT-ORF74 (Fig 1F), with CXCL1 and CXCL8 acting as agonists and CXCL10 as an inverse agonist (Table 1), as previously reported [8].

**Table 1:** Pharmacological characterization of ORF74-Rluc8.

		Binding	PLC activation		β-arrestin1 recruitment		β-arrestin2 recruitment	
		pK_i_	pEC_50_	Efficacy	pEC_50_	Efficacy	pEC_50_	Efficacy
WT	**CXCL1**	9.4 ± 0.1	8.2 ± 0.1	ago	-	-	-	-
	**CXCL8**	8.7 ± 0.1	> 7	ago	-	-	-	-
	**CXCL10**	8.6 ± 0.1	8.4 ± 0.1	inv ago	-	-	-	-
Rluc8	**CXCL1**	9.4 ± 0.1	8.1 ± 0.2	ago	8.2 ± 0.1	ago	8.5 ± 0.1^b^	ago
	**CXCL8**	8.8 ± 0.1	> 7	ago	> 7	ago	> 7	ago
	**CXCL10**	9.1 ± 0.1^a^	8.4 ± 0.2	inv ago	ND	anta	ND	anta

ORF74 binding affinities (pK_i_), potencies (pEC_50_) and efficacies to modulate ORF74-mediated PLC activity and recruit β-arrestins of CXCL1, CXCL8 or CXCL10. Values are presented as mean ± SEM of at least three independent experiments each performed in triplicate. Significant differences between pK_i_ values of WT-ORF74 (WT) and ORF74-Rluc8 (Rluc8) for corresponding chemokines (^a^ p ≤ 0.01) or between pEC_50_ values of β-arrestin1 and β-arrestin2 (^b^ p<0.05) were determined by a Student t test. ND = not determined, ago = agonist, inv ago = inverse agonist and anta = antagonist.

**Fig 2:**
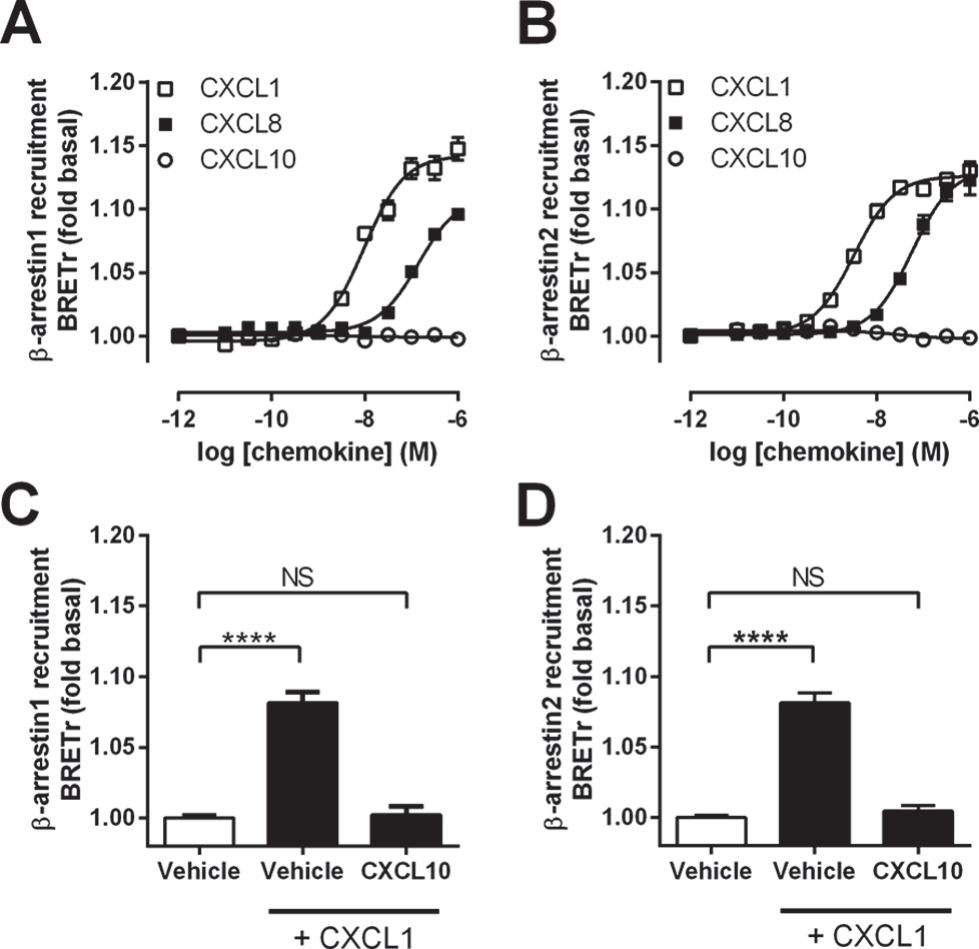
ORF74 recruits β-arrestin1 and β-arrestin2 in response to human chemokines. HEK293T cells co-expressing ORF74-Rluc8 and β-arrestin1-eYFP (A, C) or β-arrestin2-eYFP (B, D) were stimulated with increasing concentrations of CXCL1 (open squares), CXCL8 (filled squares) or CXCL10 (open circles) (A, B) or co-stimulated with CXCL1 and CXCL10 (C, D). β-arrestin recruitment to the receptor was measured as an increase in BRET ratio (BRETr). Data are shown as fold over basal and represent the mean of pooled data from at least three independent experiments each performed in triplicate. Error bars indicate SEM values. Significant differences between vehicle and chemokine-stimulation were determined by one-way ANOVA followed by a Bonferroni test (**** p ≤ 0.0001). NS = not significant.

To study β-arrestin recruitment, HEK293T cells co-expressing ORF74-Rluc8 and β-arrestin1/2 eYFP [14, 27] were stimulated with increasing concentrations chemokine. CXCL1 induced both β-arrestin1 (Fig 2A) and β-arrestin2 (Fig 2B) recruitment to ORF74 with a 2.2-fold higher potency for β-arrestin2 (Table 1). CXCL8 induced β-arrestin1 and β-arrestin2 recruitment to ORF74 with a potency at least 16-fold lower as compared to CXCL1, whereas CXCL10 displayed neutral efficacy (Fig 2A and 2B). Although CXCL1 and CXCL8 reached similar levels for β-arrestin2 recruitment, CXCL8 did not reach the levels obtained with CXCL1 for β-arrestin1 recruitment at 1 μM. However, due to its low potency, CXCL8 did not reach maximum β-arrestin recruitment at high concentrations and therefore it is not possibly to accurately determine potency and efficacy. Hence, it is unknown whether CXCL8 shows actual differences in efficacy or in potency between β-arrestin1 and β-arrestin2 recruitment. As expected, 100 nM CXCL10 antagonized β-arrestin1/2 recruitment to ORF74 in response to 10 nM CXCL1 (Fig 2C and 2D). Since CXCL10 displayed no efficacy in β-arrestin recruitment (Table 1), this might suggest that ORF74 does not constitutively recruit β-arrestins. Therefore saturation BRET experiments were performed to quantify basal (agonist-independent) β-arrestin recruitment to ORF74. To this end, a single concentration of BRET donor DNA was co-transfected with increasing concentrations of BRET acceptor DNA [28]. The BRET signal remained unchanged with increasing β-arrestin1-eYFP/ORF74-Rluc8 (S1A Fig) or β-arrestin2-eYFP/ORF74-Rluc8 (S1B Fig) ratios instead of a saturated increase representing a specific interaction with β-arrestins as observed for the dopamine D_2_ receptor [29]. Furthermore, no difference in BRET was observed between ORF74-Rluc8 and ORF74-ST/A2-Rluc8 (see below), indicating that ORF74 is unable to constitutively recruit β-arrestins.

### The G protein-uncoupled mutant ORF74-R^3.50^A recruits β-arrestin

**Fig 3:**
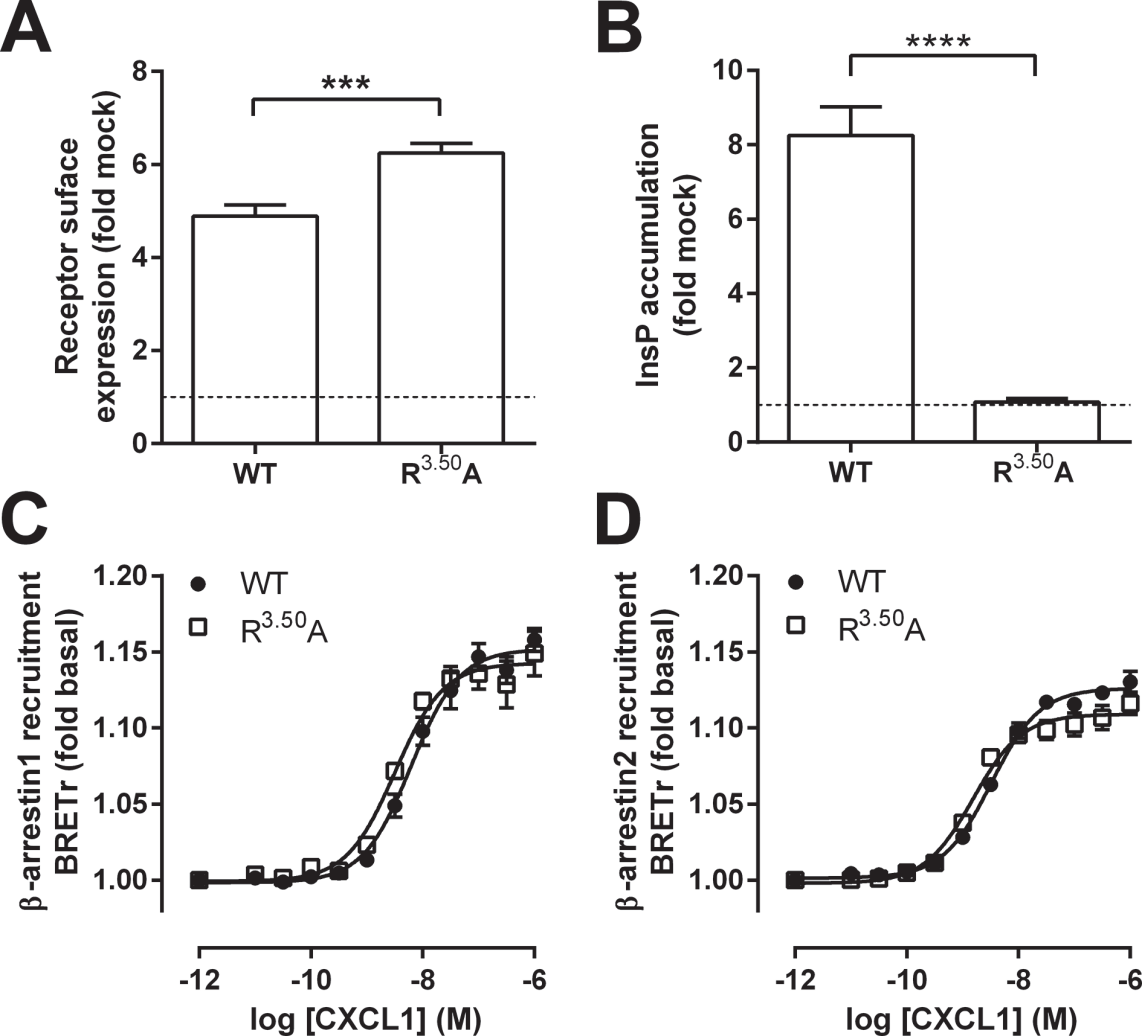
Characterization and β-arrestin recruitment to ORF74-R^3.50^A. (A, B) HEK293T cells were transiently transfected with WT-ORF74 (WT), ORF74-R^3.50^A (R^3.50^A) or empty vector DNA (mock-transfected). Relative receptor expression at the cell surface was determined by ELISA (A) and constitutive activation of PLC was determined by measuring InsP accumulation (B). Data are presented as fold over mock-transfected cells (dotted line). (C, D) HEK293T cells expressing ORF74-Rluc8 (WT) (filled circles) or ORF74-R^3.50^A-Rluc8 (R^3.50^A) (open squares) in combination with β-arrestin1-eYFP (C) or β-arrestin2-eYFP (D) were stimulated with increasing concentrations of CXCL1. Data are shown as fold over basal. All data are represented as the mean of pooled data from at least three independent experiments each performed in triplicate and error bars indicate SEM values. Statistical differences of cell surface expression (A) or constitutive PLC activation (B) between WT-ORF74 and ORF74-R^3.50^A were determined by a Student t test (**** p ≤ 0.0001, *** p ≤ 0.001).

The conserved DRY motif (Asp-Arg-Tyr), located at the boundary between transmembrane domain 3 and intracellular loop 2 of GPCRs, is required for G protein activation. Mutation of the highly conserved R^3.50^ (Ballesteros-Weinstein numbering [17]) impairs G protein-dependent signaling of many GPCRs [30], including ORF74 [31–33]. ORF74-R^3.50^A was expressed at the cell surface at levels 1.3-fold higher compared to WT-ORF74 as determined by ELISA (Fig 3A), but completely lost its ability to constitutively activate PLC (Fig 3B) and showed no CXCL1- or CXCL8-agonism (S2 Fig) as previously reported [32]. Although unable to activate PLC signaling, ORF74-R^3.50^A recruited both β-arrestin1 (Fig 3C) and β-arrestin2 (Fig 3D) in response to CXCL1 with potencies (pEC_50_ values are 8.5 ± 0.1 and 8.8 ± 0.1, respectively) that did not significantly differ from WT-ORF74 (see Table 1).

### Serine and threonine residues in the C-tail of ORF74 are essential for β-arrestin recruitment but not for G protein activation

**Fig 4:**
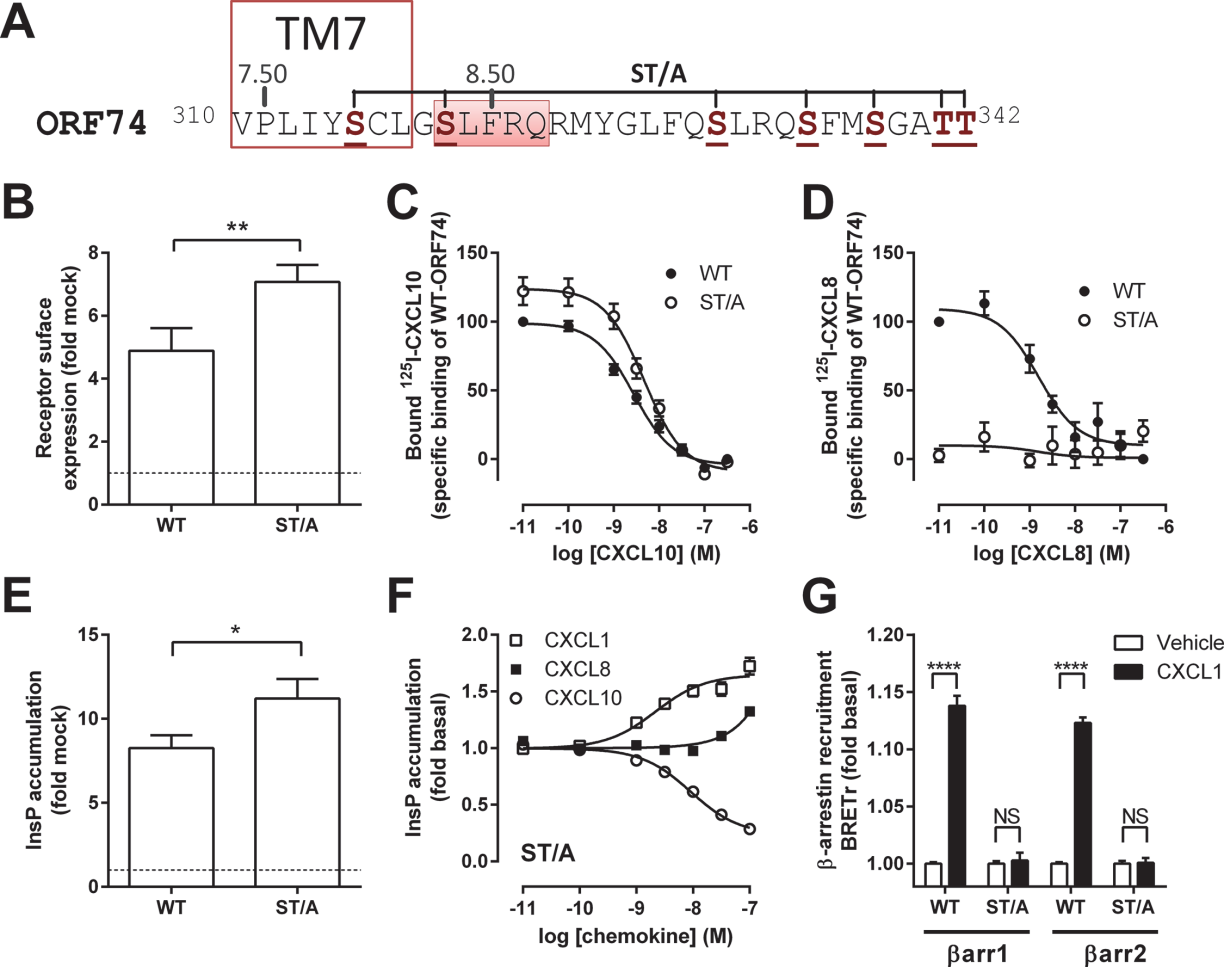
Characterization and β-arrestin recruitment to ORF74-ST/A. (A) Schematic representation of the C-tail of ORF74, starting at the conserved VPxxY-motif in TM7. Serine and threonine residues mutated to alanine in ORF74-ST/A are shown in bold brown. The location of TM7 (delineated) and helix 8 (marked red) are based on the CCR5 crystal structure (PDB-code 4MBS) [35]. (B-F) HEK293T cells were transiently transfected with WT-ORF74 (WT) (B-E) or ORF74-ST/A (ST/A) (B-F) or empty vector DNA (mock-transfected) (B, E). (B) Relative receptor expression at the cell surface was determined by ELISA. Binding of ^125^I-CXCL10 (C) or ^125^I-CXCL8 (D) to intact HEK293T cells was measured in the presence of increasing concentrations unlabeled homologous chemokines. Constitutive (E) or chemokine-induced (F) activation of PLC was determined by measuring InsP accumulation. (G) HEK293T cells expressing ORF74-Rluc8 (WT) or ORF74-ST/A-Rluc8 (ST/A) in combination with β-arrestin1-eYFP (βarr1) or β-arrestin2-eYFP (βarr2) were vehicle-stimulated (white bars) or stimulated with 300 nM CXCL1 (black bars) before measurement of BRET. Data are presented as fold over mock-transfected cells (dotted line) (B, E), percentage of specific binding (C, D) or fold over basal (F, G). All data are represented as the mean of pooled data from at least three independent experiments each performed in triplicate and error bars indicate SEM values. Statistical differences of cell surface expression (B) or constitutive PLC activation (E) between WT-ORF74 and ORF74-ST/A or between vehicle- and corresponding CXCL1-treated cells (G) were determined by a Student t test (**** p ≤ 0.0001, ** p ≤ 0.01, * p ≤ 0.05). NS = not significant.

Phosphorylation of S and T residues in the C-tail is essential for β-arrestin recruitment to some GPCRs, but not to others [34]. In order to investigate whether these putative phosphorylation sites are important for β-arrestin1/2 recruitment to ORF74, all S and T residues in the C-tail of ORF74 were Ala-substituted (ORF74-ST/A) (Fig 4A). Cell surface expression (Fig 4B) and the number of CXCL10-binding sites (Fig 4C) of ORF74-ST/A were 1.4-fold and 1.2-fold higher, respectively, as compared to WT-ORF74. ORF74-ST/A showed an unchanged affinity for CXCL10 (pK_i_ = 8.4 ± 0.2) as compared to WT-ORF74 (see Table 1). In contrast, CXCL8 binding to ORF74-ST/A was dramatically reduced (Fig 4D). Constitutive signaling of ORF74-ST/A to PLC was 1.4-fold higher as compared to WT-ORF74 (Fig 4E), which is in line with the increased cell surface expression. CXCL1 stimulated ORF74-ST/A-induced PLC activation (Fig 4F) with a 3.2-fold higher potency (pEC_50_ = 8.7 ± 0.1), as compared to WT-ORF74 (see Table 1). In contrast, the potency of CXCL10 to inhibit constitutive PLC activation was 2.5-fold lower for ORF74-ST/A (pEC_50_ = 8.0 ± 0.2) as compared to WT-ORF74 (see Table 1). Surprisingly, despite undetectable CXCL8 binding to ORF74-ST/A at 100 pM, CXCL8 stimulated ORF74-ST/A-induced PLC activation with low affinity (pEC_50_ > 7), indicating that CXCL8 is still able to bind to ORF74-ST/A at high concentrations. Ala-substitution of all S and T residues in the C-tail of ORF74 totally abolished β-arrestin1 or β-arrestin2 recruitment in response to CXCL1 (Fig 4G). The lack of β-arrestin1/2 recruitment to ORF74-ST/A was not due to the inability of chemokines to bind to this mutant as shown by chemokine-induced PLC activation of ORF74-ST/A.

### Distal serine and threonine residues in the C-tail of ORF74 interact with β-arrestin

**Fig 5:**
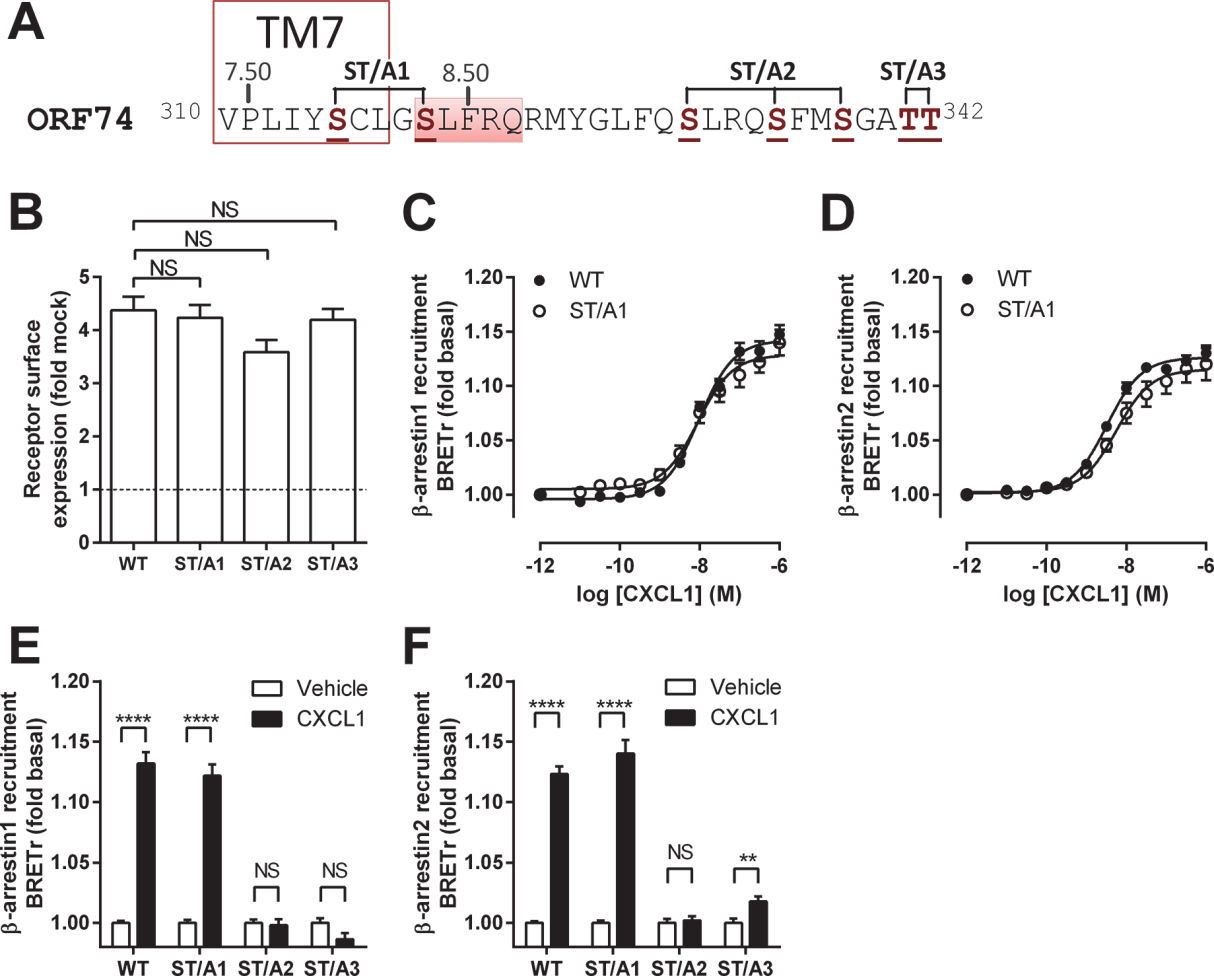
Serines and threonines at the distal end of the C-tail are essential for β-arrestin recruitment. (A) Schematic representation of the C-tail of ORF74, starting at the conserved VPxxY-motif in TM7. Serine and threonine residues mutated to alanine are shown in bold brown and clustered to indicate the different ORF74-ST/A mutants (ST/A1, ST/A2 and ST/A3). The location of TM7 (delineated) and helix 8 (marked red) are based on the CCR5 crystal structure (PDB-code 4MBS) [35]. (B) HEK293T cells were transiently transfected with ORF74-Rluc8 (WT), ORF74-ST/A1-Rluc8 (ST/A1), ORF74-ST/A2-Rluc8 (ST/A2) or ORF74-ST/A3-Rluc8 (ST/A3) or empty vector DNA (mock-transfected) and receptor cell surface expression was determined by ELISA. (C-F) HEK293T cells expressing ORF74-Rluc8 (WT) or one of the Rluc8-tagged ORF74-ST/A mutants in combination with β-arrestin1-eYFP (C, E) or β-arrestin2-eYFP (D, F) were treated with increasing concentrations CXCL1 (C, D) or were vehicle-stimulated (white bars) or stimulated with 300 nM CXCL1 (black bars) (E, F) before measurement of BRET. Data are shown as the mean of pooled data from three independent experiments each performed in triplicate. Data is presented as fold over mock-transfected cells (dotted line) (B) or fold over basal (C-F) and error bars indicate SEM values. Statistical differences between ORF74 WT and mutant cell surface expression (B) or difference between vehicle- and corresponding CXCL1-treated cells (E, F) were determined by one-way ANOVA followed by a Bonferroni test (B) or a Student t test (E, F), respectively (**** p ≤ 0.0001, ** p ≤ 0.01). NS = not significant.

To identify which S/T residues are involved in β-arrestin recruitment, Ala-substitutions were introduced in three different segments of the C-tail of ORF74 (ST/A1, ST/A2 and ST/A3) (Fig 5A). The Rluc8-tagged ORF74 mutants were expressed at the cell surface at levels similar to WT-ORF74-Rluc8, as determined by ELISA (Fig 5B). ORF74-ST/A1 recruited β-arrestin1 (Fig 5C) and β-arrestin2 (Fig 5D) in response to CXCL1 with potencies (pEC_50_ = 8.0 ± 0.1 and 8.3 ± 0.1, respectively) that did not significantly differ from WT-ORF74 (see Table 1). On the contrary, ORF74-ST/A2 completely lost its ability to recruit β-arrestin1 (Fig 5E) and β-arrestin2 (Fig 5F) in response to CXCL1. ORF74-ST/A3 was unable to recruit β-arrestin1 (Fig 5E), but showed a small increase in CXCL1-induced β-arrestin2 recruitment (Fig 5F). These results show that the two distal S/T clusters, but not the two proximal S residues in the C-terminal region of ORF74, are essential for β-arrestin1/2 recruitment.

**Fig 6:**
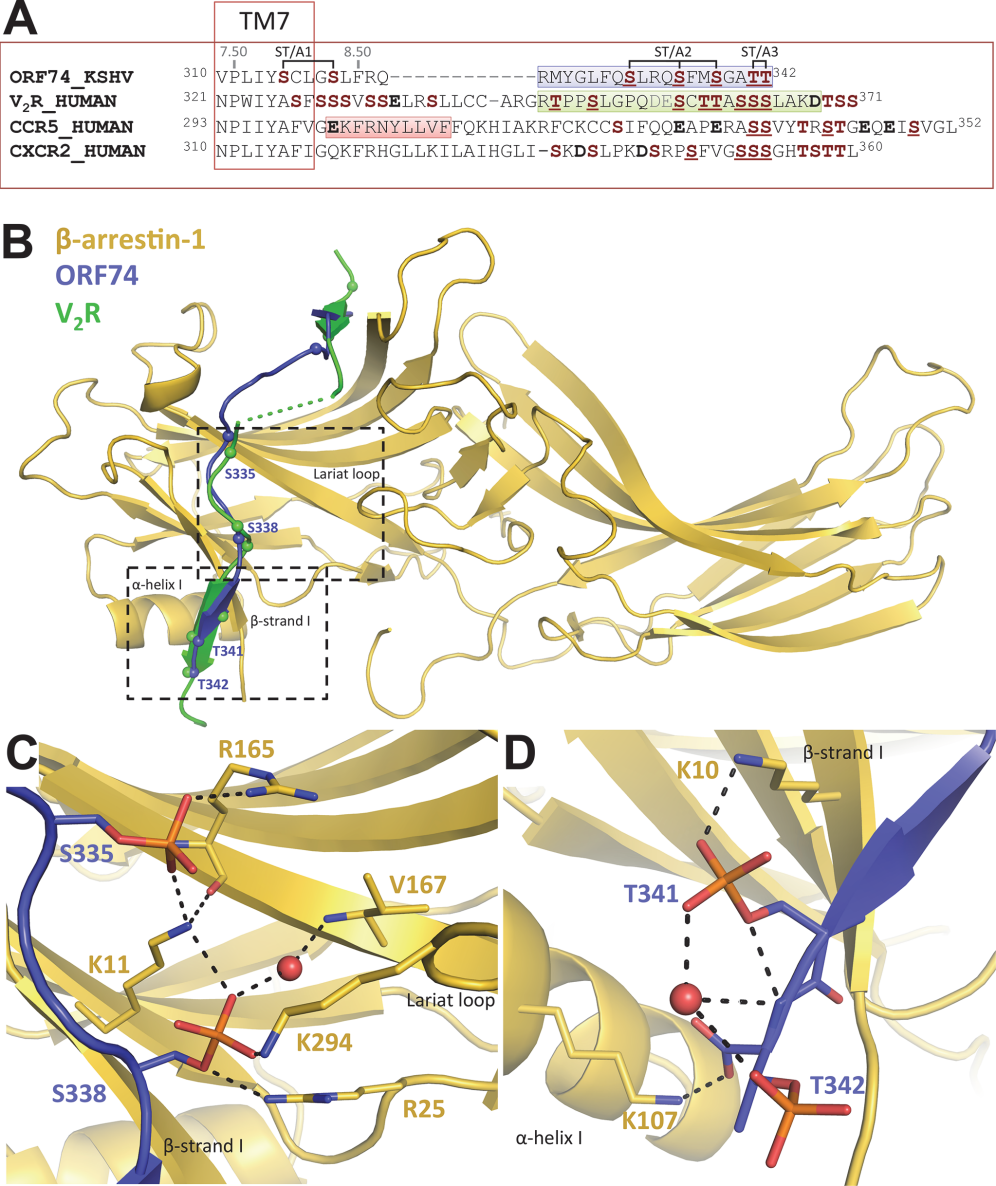
Homology model of the phosphorylated ORF74 C-tail bound to β-arrestin1. (A) Sequence alignment of V_2_R, CCR5, CXCR2, and ORF74 with a focus on serine (S) and threonine (T) residues (bold, brown) and aspartate (D) and glutamate (E) residues (bold). Underlined S/T residues are experimentally determined to be phosphorylated [37–45] or are phosphorylated in the active β-arrestin1 crystal structure [20]. The location of TM7 (delineated) and helix 8 (marked red) are based on the CCR5 crystal structure (PDB-code 4MBS) (35). Residues of the V_2_R-peptide solved in the active β-arrestin1 crystal structure are marked light green (the grey residues were not solved in the crystal structure), and the final 19 C-terminal residues of ORF74 that were used to build the model are marked light blue. (B) 3D model of the C-tail of ORF74 (blue) based on the crystal structure of the C-tail of V_2_R (green) in β-arrestin1 (gold) (PDB-code 4JQI). Spheres indicate the Cα-atoms of S/T residues of V_2_R and ORF74. A detailed view of S335/S338 (C) and T341/T342 (D) interacting with β-arrestin1 highlighting interactions between phosphorylated S/T residues in ORF74 with several key residues [36] in β-arrestin1 (i.e. K10, K11, K107, R165, K294). Dashed lines indicate H-bonds or ionic interactions. β-arrestin1-carbon, ORF74-carbon, nitrogen, oxygen, and phosphate atoms are colored gold, slate, blue, red, and orange respectively. Water molecules are depicted as red spheres.

Alignment of the C-termini of ORF74 and V2 vasopressin receptor (V_2_R) shows spatial conservation of S/T residues that are involved in the interaction between V_2_R and active β-arrestin1 in the recently published crystal structure (Fig 6A) [20]. Indeed, our homology model of the ORF74 C-tail bound to β-arrestin1 indicates that the four distal S/T residues of ORF74 bind the active β-arrestin1 structure in a similar fashion as the phosphorylated S/T residues of the V_2_R-peptide (Fig 6B), despite the fact that the C-tail of ORF74 is 18 residues shorter (Fig 6A). S335 and S338 (mutated in ORF74-ST/A2 and aligned with S357 and T360 of the V_2_R-peptide, Fig 6A) show an elaborated H-bond/ionic-interaction network with residues from β-arrestin1 (Fig 6C). S338 interacts with K294 of the lariat loop (Fig 6C), which is proposed to be a driving force for the rearrangement of this loop during activation and subsequent stabilization of the active conformation of β-arrestin1 [20, 36]. The two threonine residues mutated in ORF74-ST/A3 align with S363 and S364 of the V_2_R-peptide (Fig 6A) and make similar interactions except for the interaction with K107 of β-arrestin1. In the model, K107 interacts with the C-tail of ORF74 (Fig 6D), but in the β-arrestin1 crystal structure K107 interacts with the side chain of the phosphorylated S364 of the V_2_R-peptide.

### β-arrestin is essential for ORF74 internalization and endocytic trafficking

β-arrestins classically mediate internalization and endocytic trafficking of GPCRs [46]. We used a recently described BRET-based approach that quantitatively measures real-time GPCR trafficking from the plasma membrane to different endosome compartments in response to agonist stimulation [18, 19, 47, 48]. To this end, ORF74-Rluc8, ORF74-ST/A2-Rluc8 or ORF74-ST/A3-Rluc8 were co-expressed in HEK293T cells with one of the four BRET acceptors localized to specific subcellular compartments: Venus-K-Ras at the plasma membrane, Venus-Rab5a in early endosomes, Venus-Rab7a in late endosomes/lysosomes, and Venus-Rab11 in recycling endosomes. All BRET acceptors were expressed at comparable protein levels, as revealed by measurements of fluorescence (S3 Fig).

**Fig 7:**
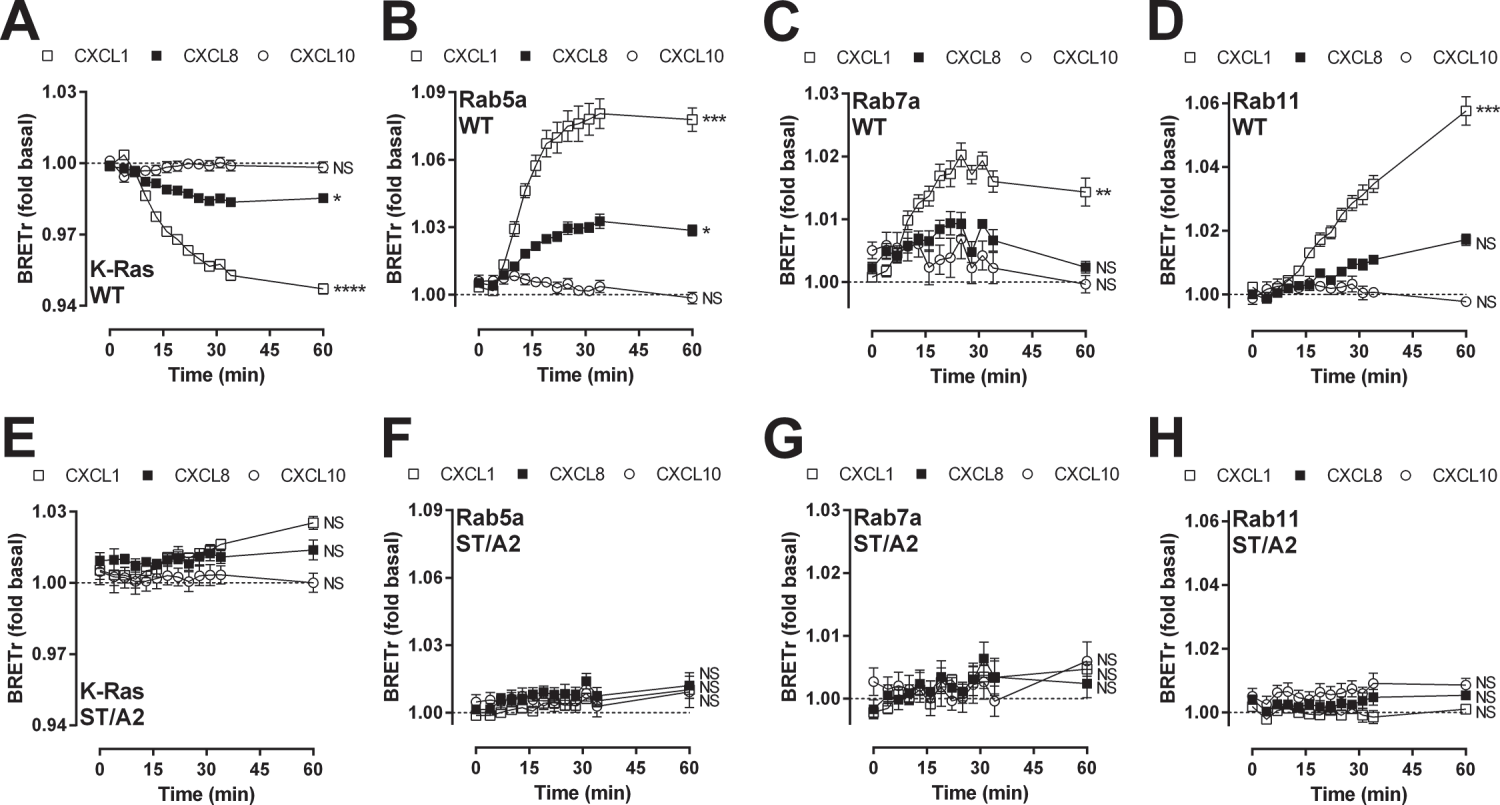
ORF74 internalizes and traffics via early, recycling and late endosomes. HEK293T cells were transiently transfected with ORF74-Rluc8 (WT) (A-D) or ORF74-ST/A2-Rluc8 (ST/A2) (E-H) in combination with Venus-K-Ras (plasma membrane marker) (A, E), Venus-Rab5a (early endosome marker) (B, F), Venus-Rab7a (late endosome/lysosome marker) (C, G) or Venus-Rab11 (recycling endosome marker) (D, H) and stimulated with CXCL1, CXCL8 or CXCL10 for indicated time and BRET was measured. Data are shown as the mean of pooled data from three independent experiments each performed in triplicate. Data is presented as fold over vehicle-stimulated cells (dotted line) and error bars indicate SEM values. Statistical differences between the area under the curve of vehicle- and corresponding CXCL1-, CXCL8- or CXCL10-treated cells (baseline = 1) were determined by one-way ANOVA followed by a Bonferroni test (**** p ≤ 0.0001, *** p≤ 0.001, ** p ≤ 0.01, * p ≤ 0.05). NS = not significant.

Stimulation with 100 nM CXCL1 rapidly and significantly decreased BRET between ORF74-Rluc8 and plasma membrane-localized Venus-K-Ras in time, indicating ORF74 internalization (Fig 7A). Simultaneously, BRET between ORF74-Rluc8 and Venus-Rab5a (Fig 7B) or Venus-Rab7a (Fig 7C) significantly increased in time with a maximum reached after 30 min. Upon CXCL1 stimulation, BRET between ORF74-Rluc8 and Venus-Rab11 significantly increased in time, but had a slower onset compared to Rab5a and Rab7a and reached maximum levels after approximately 1h (Fig 7D). CXCL8 (100 nM) induced a similar BRET change in time between ORF74-Rluc8 and Venus-K-Ras, Venus-Rab5a and Venus-Rab11 (although the latter was not significant compared to vehicle-stimulated cells), but not Venus-Rab7a. However, CXCL8 induced smaller BRET changes than CXCL1, which is in line with the observed difference in potencies of these chemokines to recruit β-arrestins (see Table 1). As expected, stimulation with 100 nM CXCL10 did not promote internalization and subsequent trafficking of ORF74 (Fig 7A–7D). The significant changes in BRET observed between ORF74-Rluc8 and Venus-K-Ras, Venus-Rab5a, Venus-Rab7a or Venus-Rab11 in response to chemokines were lacking in cells transfected with the β-arrestin1/2-uncoupled ORF74-ST/A2-Rluc8 (Fig 7E–7H) or ORF74-STA3-Rluc8 (S4A–S4D Fig). The BRET acceptor expression levels in cells co-expressing ORF74-ST/A2-Rluc8 or ORF74-ST/A3-Rluc8 were comparable to cells co-expressing ORF74-Rluc8 (S3 Fig).

**Fig 8:**
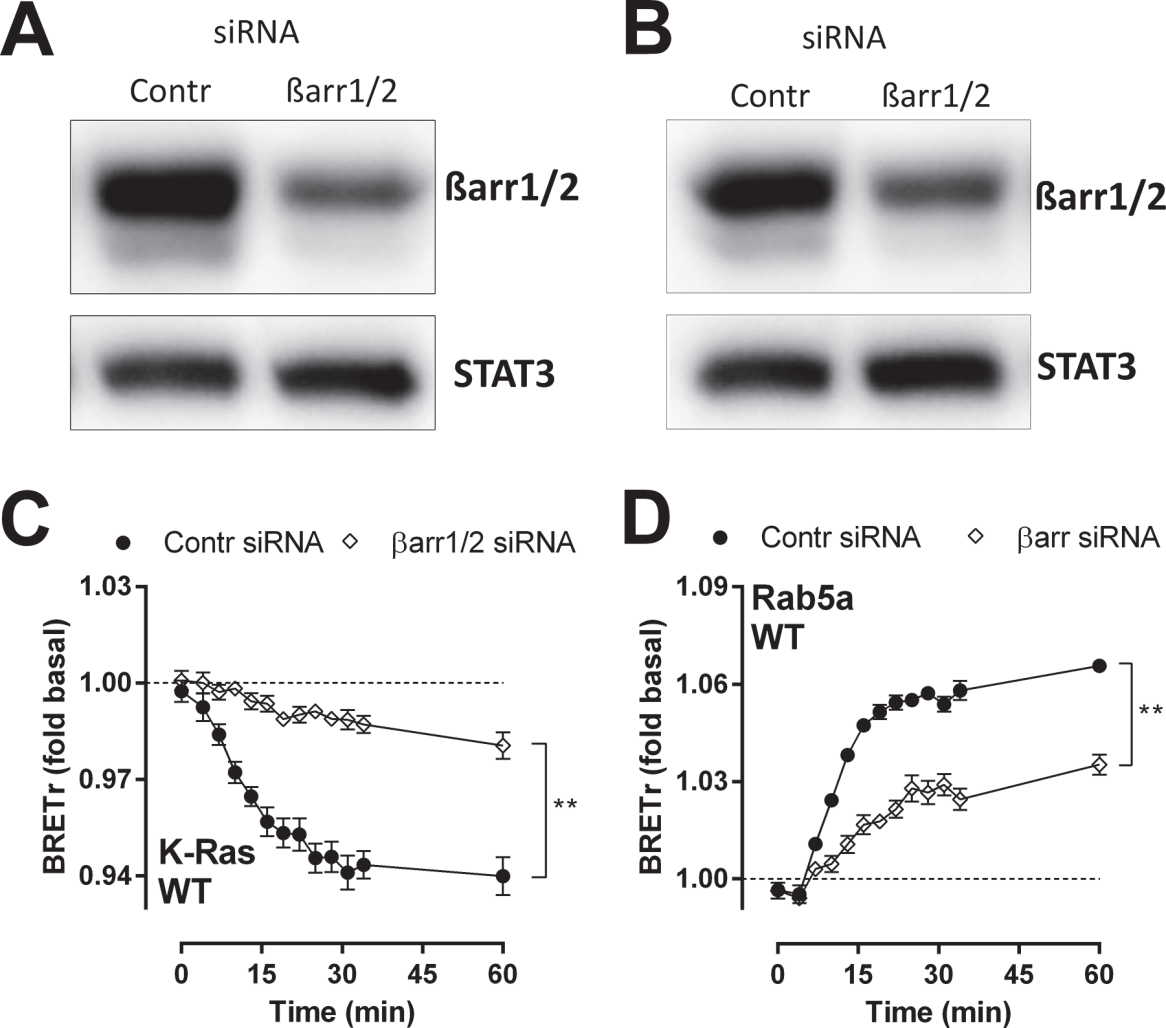
ORF74 trafficking is β-arrestin-dependent. HEK293T cells were transiently transfected with ORF74-Rluc8 and Venus-K-Ras (plasma membrane marker) (A, C) or Venus-Rab5a (early endosome marker) (B, D) in combination with control (Contr) or β-arrestin1/2 (βarr1/2) siRNA. (A, B) Downregulation of β-arrestin1/2 levels was determined by immunoblotting. STAT3 levels were determined as loading control. (C, D) Cells were stimulated with CXCL1 for indicated time and BRET was measured. Data are shown as the mean of pooled data from three independent experiments each performed in triplicate. Data is presented as fold over vehicle-stimulated cells (dotted line) and error bars indicate SEM values. Statistical differences between the area under the curve of cells treated with control or β-arrestin1/2 siRNA (baseline = 1) were determined by a Student t test (** p ≤ 0.01).

Downregulation of endogenous β-arrestin1 and β-arrestin2 (Fig 8A and 8B) inhibited the CXCL1-induced changes in BRET between ORF74-Rluc8 and Venus-K-Ras (Fig 8C) or Venus-Rab5a (Fig 8D), as compared to cells treated with control siRNA. Internalization and trafficking to early endosomes in response to CXCL1 was not completely inhibited by β-arrestin1/2 siRNA, which is probably due to the incomplete knockdown of endogenous β-arrestins (Fig 8A and 8B). These results show that β-arrestins are required for ORF74 internalization and the subsequent endocytic trafficking.

## Discussion

BRET was utilized to demonstrate for the first time that the constitutively active viral GPCR ORF74 recruits both β-arrestin1 and β-arrestin2 in response to human chemokines CXCL1 and CXCL8, but not in the absence of agonists. Furthermore, the fact that CXCL10 was unable to modulate β-arrestin1/2 recruitment to ORF74 supports the absence of constitutive β-arrestin recruitment towards ORF74. However, it cannot be excluded that CXCL10 behaves differently in G protein-independent pathways with respect to G protein-dependent pathways. On the other hand, CXCL10 fully antagonized CXCL1-induced β-arrestin1/2 recruitment. Although contradicting results have been published regarding the ability of CXCL10 to displace CXCL1 [49, 50], the observed antagonism indicates that CXCL1 and CXCL10 bind to a common population of ORF74. Recruitment of β-arrestin is independent of G protein-activation, as revealed by β-arrestin recruitment to the G protein-uncoupled mutant ORF74-R^3.50^A [31–33]. Similar conclusions were previously drawn for other receptors based on comparable mutations within the DRY motif (M_3_ muscarinic acetylcholine receptor [51]) or upon uncoupling Gα_i/o_-coupled receptors (CCR2 [52] and histamine H_4_ receptor [53]) using the Gα_i/o_ inhibitor pertussis toxin, that all retain their ability to recruit β-arrestin. Moreover, the decoy receptors CXCR7 [54, 55], and C5a receptor C5L2 [56] do not activate G protein-dependent signaling but recruit β-arrestins. In contrast, mutation of R^3.50^ in the M_1_ muscarinic acetylcholine receptor (M_1_ mAChR) or inhibition of Gα_q_ with the specific inhibitor UBO-QIC significantly reduced β-arrestin2 recruitment, indicating that β-arrestin recruitment to the M_1_ receptor is G protein-dependent [48].

The C-tail of ORF74 contains multiple serine and threonine residues that potentially function as phosphorylation sites to promote the coupling of β-arrestins. Alignment of ORF74 with sequences of crystallized class A GPCRs indicate that the first two serines following the VPxxY motif (NPxxY in the majority of GPCRs) (S315 and S319) are located in TM7 and first turn of helix 8 and are therefore unlikely to directly interact with β-arrestin. Indeed, Ala-substitution of these serines (ST/A1) did not affect CXCL1-induced β-arrestin recruitment as compared to WT-ORF74. However, Ala-substitution of the distal three serines (ORF74-ST/A2) or two threonines (ORF74-ST/A3) in the C-tail of ORF74 almost completely abolished CXCL1-induced β-arrestin recruitment. A homology model of β-arrestin1 bound to the C-tail of ORF74 rationalizes these data by showing interactions between these distal serine and threonine clusters of ORF74 with key residues in the polar core of β-arrestin1 (i.e. K10, K11, K107, R165, K294) [36]. All β-arrestin1 residues that interact with ORF74 are conserved in β-arrestin2 and can consequently not explain the differential recruitment of β-arrestin1 and β-arrestin2 to ORF74-ST/A3. On the other hand, β-arrestin2 was reported to show less preference for binding to phosphorylated GPCRs than β-arrestin1 [57] and this might explain the small but significant CXCL1-induced β-arrestin2 recruitment to ORF74-ST/A3.

ORF74 has a relatively short C-tail (21 residues after the conserved F^8.50^) compared to human chemokine receptors (38–62 residues) or other class A GPCRs [58–60] (Fig 6A). It is therefore impossible for ORF74 to interact with β-arrestin in a similar manner as GPCRs with long(er) C-tails. According to our homology model, helix 8 (residues 319–323) of ORF74 will (partially) unfold upon β-arrestin binding to allow the elongated C-tail (residues 324–342, Fig 6A) to bind to β-arrestin in a similar manner as the V_2_R-peptide in the active β-arrestin1 crystal structure [20]. NMR and CD spectroscopy studies have indicated that the secondary structure of the helix 8 region highly depends on the surrounding and does not always form a helical shape [61–63]. Moreover, the different GPCR crystal structures show high conformational diversity of this C-tail region (S5A Fig) [58]. Several crystal structures have a disordered C-tail and a very short helix 8 (δ opioid receptor) or no helix 8 (CXCR4, PAR_1_ proteinase-activated receptor and NTS_1_ neurotensin receptor) (S5B Fig). Although this structural variability in the C-tail region can be the result of crystallographic artifacts [58], this suggests that the structure of helix 8 is flexible and can adopt different conformations. The lack of a cysteine residue in the C-tail of ORF74, that acts as a potential palmitoylation target to anchor and stabilize helix 8 to the membrane in many other GPCRs [64], further supports the proposed unfolding of helix 8. Alternatively, helix 8 is maintained including the amphipathic region (residues 321–329) [65]. Consequently, only 12 residues (330–342) of the C-tail of ORF74 are free to bind to β-arrestin, thereby interacting with a smaller region of β-arrestin than the V_2_R-peptide and implying that helix 8 is also directly interacting with β-arrestin.

Interestingly, Ala-substitution of all S/T residues in the C-tail of ORF74 dramatically decreased the number of CXCL8 binding sites without influencing the affinity or number of binding sites for CXCL10. Previously we showed that ORF74-Δ24 (a C-tail deletion mutant) lost CXCL8 binding and suggested that this was the result of disruption of the interaction between helix 8 and TM7 [50]. One of the mutated serine residues in ORF74-ST/A is located in helix 8, which might indeed account for the observed decrease in CXCL8 binding. However, CXCL8 is still able to increase PLC activity in ORF74-ST/A-expressing cells. Since higher concentrations of CXCL8 are used in the functional assays (up to 100 nM) compared to the binding assays (100 pM), CXCL8 binding to ORF74-ST/A might indeed be undetectable.

Upon binding to phosphorylated GPCRs, β-arrestins classically induce receptor internalization to prevent cells from excessive receptor stimulation [66]. Internalized GPCRs are dephosphorylated in endosomes and return to the plasma membrane via recycling endosomes. Alternatively, internalized GPCRs may be sorted via late endosomes to the lysosomes for receptor degradation [66]. ORF74 rapidly internalizes in response to CXCL1 and CXCL8 and traffics via Rab5a-containing early endosomes to Rab11-containing recycling endosomes and Rab7a-containing late endosomes. The larger increase in chemokine-induced BRET between ORF74-Rluc8 and Venus-Rab5a and Venus-Rab11 in comparison to Venus-Rab7a could indicate preferred trafficking and recycling via early and recycling endosomes over degradation in lysosomes via trafficking to late endosomes, respectively, but could also be the consequence of differences in conformations and/or dipole orientations between the Venus-Rab constructs, resulting in distinct BRET efficiencies [19, 48]. A β-arrestin-uncoupled mutant of ORF74 (ORF74-ST/A2) is unable to internalize and to traffic to endocytic vesicles, indicating a role for β-arrestin in the trafficking of ORF74. A key role for β-arrestin was further confirmed by performing the BRET experiments in the presence of β-arrestin1/2 siRNA. This BRET-based assay has recently been used to quantitatively measure internalization and trafficking of the β2AR [18], the protease-activated receptor 2 (PAR_2_) [47] and the M_1_ mAChR [48]. The M_1_ mAChR traffics with similar kinetics as ORF74 to early and recycling endosomes after stimulation with carbachol, with maximum BRET between M_1_ mAChR and Rab5a after 30 minutes, whereas BRET between M_1_ mAChR and Rab11 increased until 1h [48]. Comparable to ORF74, the BRET between M_1_ mAChR and Rab7a remained close to baseline levels throughout the simulation period with carbachol [48].

In the last decade, β-arrestins have also been recognized as adaptor proteins that transduce GPCR signals independently of G proteins. To this end, β-arrestins scaffold intracellular signaling molecules, for example mitogen-activated-protein kinases (MAPKs), into a complex with the activated GPCR to facilitate their activation [67]. This β-arrestin-dependent signaling plays a role in the central nervous system, the cardiovascular system, the immune system but also in cancer [68–70]. However, the G protein-uncoupled mutant ORF74-R^3.50^A is able to recruit β-arrestins, but is not tumorigenic in transgenic mice [33] or in an allograft mouse model [31]. This suggests that β-arrestin-dependent signaling might not play a significant role in ORF74-mediated oncogenesis.

ORF74 internalization might contribute to immune evasion and consequently viral dissemination by downregulating proteins involved in anti-viral immune responses (e.g. chemokine receptors, Toll-like receptors). The Toll-like Receptor 4 (TLR4) plays a key role in the innate immunity against KSHV [71], but is indeed downregulated by ORF74. Interestingly, an ORF74 mutant that is unable to interact with clathrin-coated vesicle component adaptor protein 2 (AP-2), and is consequently expressed at higher cell surface levels [11], is unable to reduce TLR4 cell surface expression [72]. Hence, ORF74 internalization seems to contribute to viral spread by reducing TLR4 levels. Subsequent recycling of ORF74 to the cell surface might be important to maintain signaling in response to CXCL1 [73, 74].

Collectively, we show that ORF74 recruits β-arrestins in response to CXCL1 and CXCL8 and is rapidly internalized and sorted via early endosomes to recycling and late endosomes. The β-arrestin-uncoupled ORF74 mutants (ORF74-ST/A2 and ORF74-ST/A3) fail to internalize or traffic to endocytic compartments and β-arrestin1/2 siRNA inhibits ORF74 internalization, showing a key role for β-arrestin in these processes.

## Supporting Information

S1 Fig ORF74 is unable to constitutively recruit β-arrestins. HEK293T cells were transiently transfected with a constant amount of ORF74-Rluc8 (WT) (filled circles) or ORF74-ST/A2-Rluc8 (ST/A2) (open circles) and increasing amount of β-arrestin1-eYFP (A) or β-arrestin2-eYFP (B). The BRET ratio (BRET/Rluc8) was measured as a function of increasing eYFP/Rluc8 ratio and is plotted as fold Rluc only (determined in the absence of β-arrestin1/2-eYFP). Pooled data from six independent experiments each performed in triplicate is shown.
(TIF)

S2 Fig ORF74-R^3.50^A is unable to activate PLC in response to chemokines. HEK293T cells were transiently transfected with ORF74-R^3.50^A or empty vector DNA (mock-transfected) and activation of PLC was determined by measuring InsP accumulation. Data are presented as fold over mock-transfected cells (dotted line) and all data are represented as the mean of pooled data from three independent experiments each performed in triplicate and error bars indicate SEM values. Statistical differences of PLC activation between vehicle- and chemokine-treated cells were determined by one-way ANOVA followed by a Bonferroni test. NS = ‘not significant’.
(TIF)

S3 Fig Comparable expression levels of BRET acceptors when co-expressed with Rluc-tagged ORF74-(mutants). HEK293T cells were transiently transfected with ORF74-Rluc8 (WT) (white bars), ORF74-ST/A2-Rluc8 (black bars) or ORF74-ST/A3-Rluc8 (shaded bars) in the absence (mock-transfected cells, dotted line) or presence of Venus-K-Ras (plasma membrane marker), Venus-Rab5a (early endosome marker), Venus-Rab7a (late endosome/lysosome marker) or Venus-Rab11 (recycling endosome marker) and fluorescence was measured. The mean ± SEM of a representative experiment performed in triplicate are shown and data is presented as fold over mock-transfected cells (dotted line). The experiment was repeated two times.
(TIF)

S4 Fig Threonines at the distal end of the C-tail are essential for endocytic trafficking of ORF74. HEK293T cells were transiently transfected with ORF74-ST/A3-Rluc8 in combination with Venus-K-Ras (plasma membrane marker) (A), Venus-Rab5a (early endosome marker) (B), Venus-Rab7a (late endosome/lysosome marker) (C), Venus-Rab11 (recycling endosome marker) (D) and stimulated with CXCL1, CXCL8 or CXCL10 for indicated time and BRET was measured. Data are shown as the mean of pooled data from three independent experiments each performed in triplicate. Data is presented as fold over vehicle-stimulated cells (dotted line) and error bars indicate SEM values. Statistical differences between the area under the curve of vehicle- and corresponding CXCL1-, CXCL8 or CXCL10-treated cells (baseline = 1) were determined by one-way ANOVA followed by a Bonferroni test. NS = not significant.
(TIF)

S5 Fig Helix 8 is not always present in class A GPCR crystal structures and shows variability in orientation and length. (A) Sequence overview highlighting the secondary structure of TM7 (marked gray) and helix 8 (marked red) in all crystal structures. The sequences shown are from the crystallographic constructs with indicated PDBs. Grey residues were not resolved in the crystal structure. (B) An alignment of the end of TM7 and the full C-tail of a single crystal structure per crystallized class A GPCR based on the conserved NPxxY motif in TM7.
(TIF)
