# Supplementary figures and images for: The Viral G Protein-Coupled Receptor ORF74 Hijacks β-Arrestins for Endocytic Trafficking in Response to Human Chemokines
Source: PLoS One. 2015 Apr 20;10(4):e0124486. doi: 10.1371/journal.pone.0124486 (PMC4403821; doi:10.1371/journal.pone.0124486)

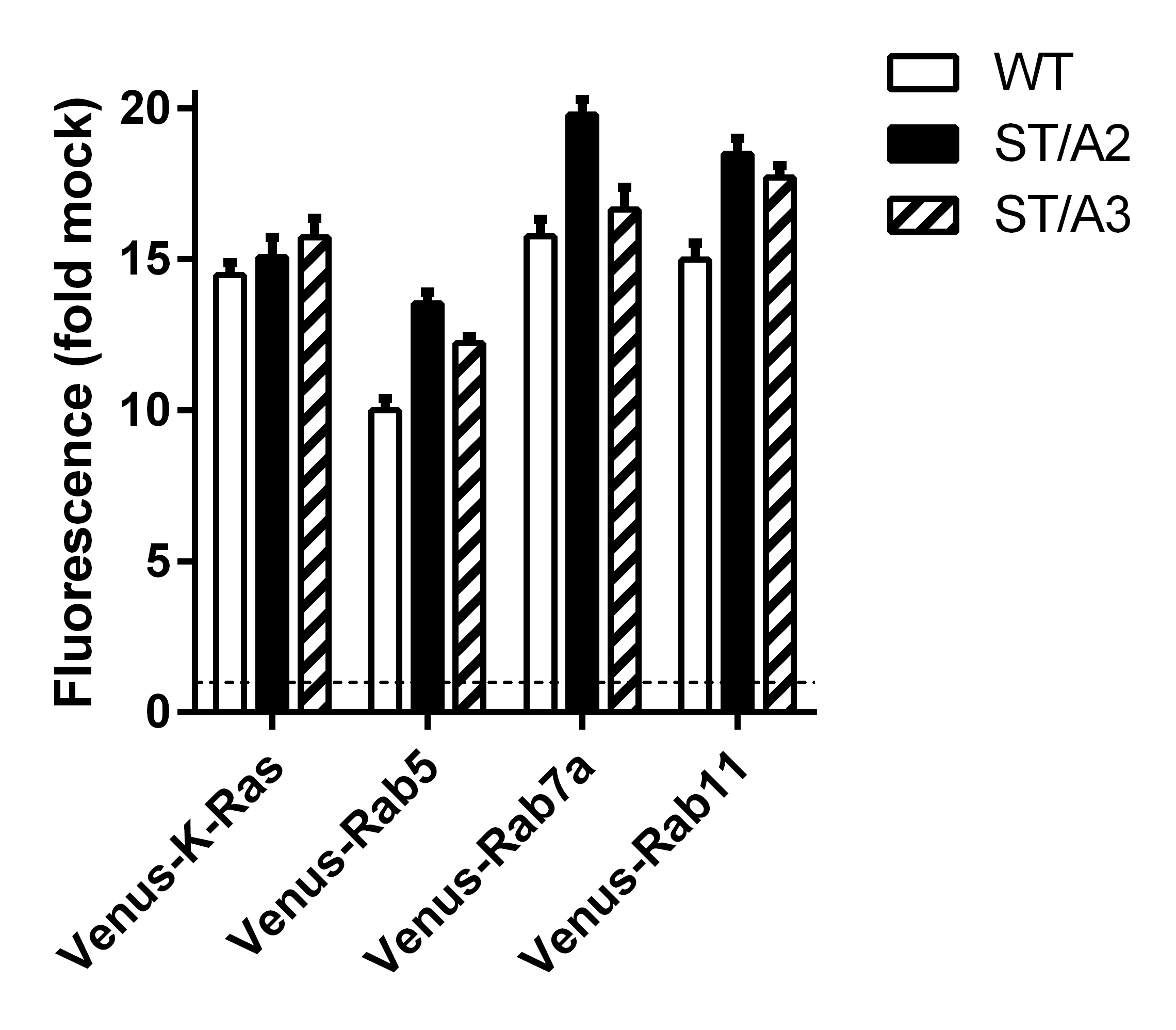

Supplement: S3 Fig — HEK293T cells were transiently transfected with ORF74-Rluc8 (WT) (white bars), ORF74-ST/A2-Rluc8 (black bars) or ORF74-ST/A3-Rluc8 (shaded bars) in the absence (mock-transfected cells, dotted line) or presence of Venus-K-Ras (plasma membrane marker), Venus-Rab5a (early endosome marker), Venus-Rab7a (late endosome/lysosome marker) or Venus-Rab11 (recycling endosome marker) and fluorescence was measured. The mean ± SEM of a representative experiment performed in triplicate are shown and data is presented as fold over mock-transfected cells (dotted line). The experiment was repeated two times. (TIF) [file pone.0124486.s003.tif]

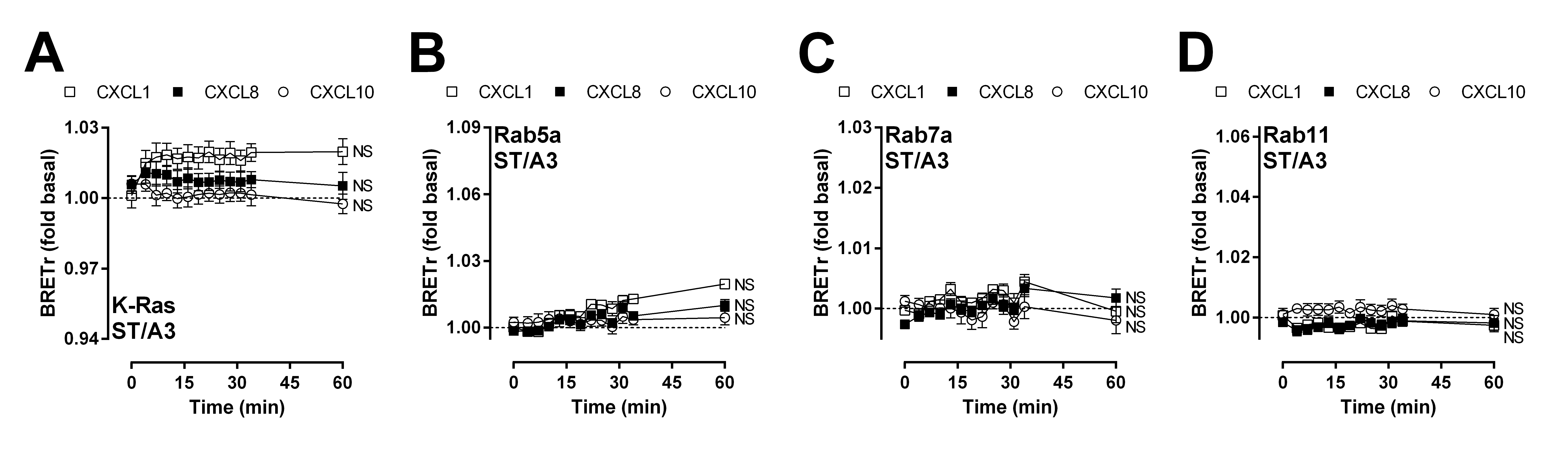

Supplement: S4 Fig — HEK293T cells were transiently transfected with ORF74-ST/A3-Rluc8 in combination with Venus-K-Ras (plasma membrane marker) (A), Venus-Rab5a (early endosome marker) (B), Venus-Rab7a (late endosome/lysosome marker) (C), Venus-Rab11 (recycling endosome marker) (D) and stimulated with CXCL1, CXCL8 or CXCL10 for indicated time and BRET was measured. Data are shown as the mean of pooled data from three independent experiments each performed in triplicate. Data is presented as fold over vehicle-stimulated cells (dotted line) and error bars indicate SEM values. Statistical differences between the area under the curve of vehicle- and corresponding CXCL1-, CXCL8 or CXCL10-treated cells (baseline = 1) were determined by one-way ANOVA followed by a Bonferroni test. NS = not significant. (TIF) [file pone.0124486.s004.tif]

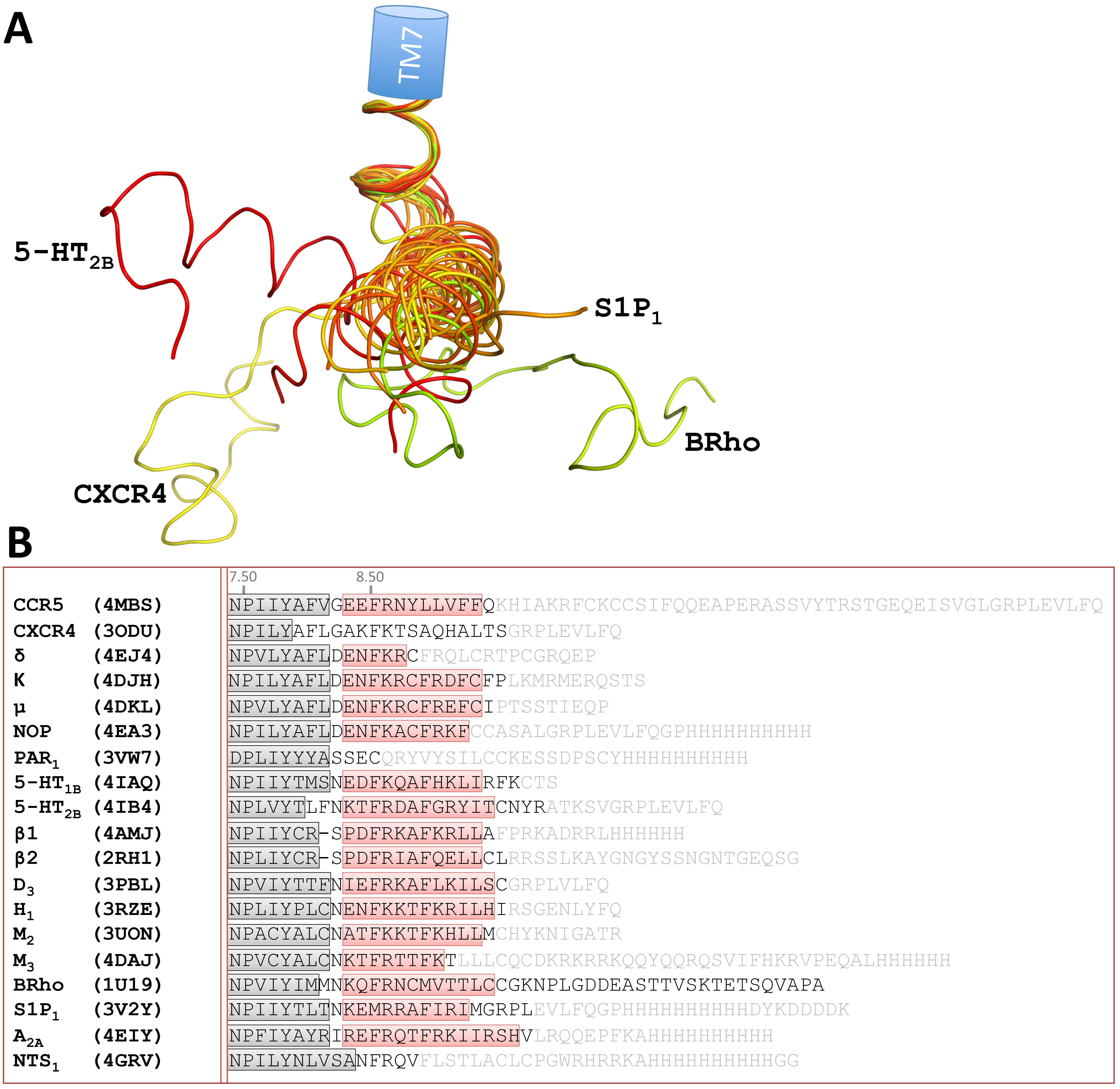

Supplement: S5 Fig — (A) Sequence overview highlighting the secondary structure of TM7 (marked gray) and helix 8 (marked red) in all crystal structures. The sequences shown are from the crystallographic constructs with indicated PDBs. Grey residues were not resolved in the crystal structure. (B) An alignment of the end of TM7 and the full C-tail of a single crystal structure per crystallized class A GPCR based on the conserved NPxxY motif in TM7. (TIF) [file pone.0124486.s005.tif]
